# Inducer-free cellulase production system based on the constitutive expression of mutated XYR1 and ACE3 in the industrial fungus *Trichoderma* *reesei*

**DOI:** 10.1038/s41598-022-23815-4

**Published:** 2022-11-14

**Authors:** Toshiharu Arai, Sakurako Ichinose, Nozomu Shibata, Hiroshi Kakeshita, Hiroshi Kodama, Kazuaki Igarashi, Yasushi Takimura

**Affiliations:** Biological Science Research, Kao Corporation, 1334 Minato, Wakayama, Wakayama 640‑8580 Japan

**Keywords:** Biotechnology, Microbiology

## Abstract

*Trichoderma* *reesei* is a widely used host for producing cellulase and hemicellulase cocktails for lignocellulosic biomass degradation. Here, we report a genetic modification strategy for industrial *T.* *reesei* that enables enzyme production using simple glucose without inducers, such as cellulose, lactose and sophorose. Previously, the mutated XYR1^V821F^ or XYR1^A824V^ was known to induce xylanase and cellulase using only glucose as a carbon source, but its enzyme composition was biased toward xylanases, and its performance was insufficient to degrade lignocellulose efficiently. Therefore, we examined combinations of mutated XYR1^V821F^ and constitutively expressed CRT1, BGLR, VIB1, ACE2, or ACE3, known as cellulase regulators and essential factors for cellulase expression to the *T.* *reesei* E1AB1 strain that has been highly mutagenized for improving enzyme productivity and expressing a ß-glucosidase for high enzyme performance. The results showed that expression of ACE3 to the mutated XYR1^V821F^ expressing strain promoted cellulase expression. Furthermore, co-expression of these two transcription factors also resulted in increased productivity, with enzyme productivity 1.5-fold higher than with the conventional single expression of mutated XYR1^V821F^. Additionally, that productivity was 5.5-fold higher compared to productivity with an enhanced single expression of ACE3. Moreover, although the DNA-binding domain of ACE3 had been considered essential for inducer-free cellulase production, we found that ACE3 with a partially truncated DNA-binding domain was more effective in cellulase production when co-expressed with a mutated XYR1^V821F^. This study demonstrates that co-expression of the two transcription factors, the mutated XYR1^V821F^ or XYR1^A824V^ and ACE3, resulted in optimized enzyme composition and increased productivity.

## Introduction

Replacing fossil fuels with environmentally clean and renewable energy sources is essential to building a sustainable society. Lignocellulosic biomass is the most abundant sustainable feedstock for biorefinery and biofuel production. Therefore, effectively utilizing lignocellulosic biomass could assist in addressing the problem^1,2^.

In order to use lignocellulosic biomass as raw material, it is necessary to hydrolyze lignocellulosic biomass into simple sugars using cellulase and hemicellulase. Extracellular cellulases and hemicellulases for lignocellulosic biomass degradation are produced mainly by filamentous fungi^3^. Especially the filamentous fungus *Trichoderma* *reesei* (teleomorph *Hypocrea* *jecorna*) is a well-known microorganism that produces large quantities of mixed extracellular cellulase and hemicellulase enzymes for the degradation of lignocellulosic biomass^4,5^. It has been expected to be the mainstay of commercial cellulase production, considering reports that industrial strains of *T.* *reesei* can be used to obtain up to over 80 g/L of extracellular protein^4,6–8^. Nevertheless, enzymatic hydrolysis of lignocellulosic biomass requires large amounts of lignocellulolytic enzymes, and the high cost of the enzymes is recognized as a major bottleneck in the production of lignocellulosic biofuels^9–12^.

One of the best solutions is to enhance enzyme production capacity and use inexpensive carbon sources such as glucose. Hence, the regulatory mechanisms of cellulase expression must be well understood. However, the cellulase expression is complexly regulated by several transcription factors (TFs), which may directly or indirectly influence cellulase gene expression^13^. Therefore, the specific roles of various factors and their complete regulatory network is not yet fully understood. Previous studies have demonstrated the role of various cellulase regulators. While XYR1, HAP2/3/5, ACE2, VEL1, ACE3, ARE1, CRZ1, STR1, and VIB1 are positive regulators, CRE1, ACE1, RCE1, CTF1, LAE1, and PAC1 are identified as negative regulators^14,15^. Cellulase gene expression requires the release of carbon catabolite repression (CCR)^16^. Therefore, modification of cellulase regulators by genetic technology is considered effective.

The transcriptional repressor CRE1 is a key regulator of the CCR and is known to indirectly inhibit cellulase expression in filamentous fungi in the presence of glucose^16^. In *Trichoderma*, truncation^17^, deletion^18^, or multisite-directed mutagenesis^19^ of *cre1* alleviates the CCR. Thus, it is possible to markedly enhance the cellulase gene expression in the presence of various carbon sources such as glucose, lactose, sophorose, and cellulose^20–23^. In particular, either deletion or truncation of *cre1* leads to partial de-repression of cellulase and hemicellulase when CRE1-mutated strains are cultured in a glucose-containing medium^16–18,24^. However, in a medium containing only glucose without inducing sugars, the CRE1 mutated strain cannot completely release the CCR. Thus, it either represses expression or neither represses nor activates cellulase or hemicellulase, resulting in low cellulase production. In addition, the repressors *ace1*^25^ and *rce1*^26^, as well as *cre1*, can be deleted to improve cellulase gene expression levels, but inducers are assumed to be necessary for expression.

In contrast, XYR1, a positive regulator required for expressing most cellulase and hemicellulase genes, led to the potential to express these genes without inducers by modification. XYR1 has a Zn(II)_2_Cys_6_-type DNA-binding domain, which binds directly to the upstream regions of the cellulase/hemicellulase genes^27,28^. Disruption of *xyr1* leads to a cellulase-negative phenotype^29^, indicating that it is an essential factor for cellulase induction. XYR1 promotes increased cellulase gene expression under conditions of constitutive overexpression, whereas it does not increase cellulase production in media with glucose as the sole carbon source^12^. Engineering of TFs has also been implemented to increase cellulase expression. Despite artificial TFs that fuse XYR1 with other transcription factors that have been developed and attempted to produce cellulase constitutively using glucose as the sole carbon source, the protein productivity has not led to induced production^30,31^. The mutated XYR1^V821^^F^ or XYR1^A824V^ expressing strain expressed xylanase and cellulase even in glucose as the sole carbon source and improved protein productivity^8,32,33^. Nevertheless, the enzymes induced by the mutated XYR1^V821F^ or XYR1^A824V^ were not sufficient for the efficient degradation of lignocellulose because of their high xylanase and low cellulase composition^32,33^. Therefore, to produce cellulase/hemicellulases at levels similar to the induced state even in media with glucose as the sole carbon source, we presumed that it is necessary to add factors that activate cellulase expression working in cooperation with XYR1, and we searched for cooperative factors to express with mutated XYR1^V821F^.

The transcription activators ACE2^34^, ACE3^35^, and VIB1^36^, were putative cooperative factors. In addition, CRT1^37,38^, a cellulose-responsive transporter involved in the regulation of *xyr1* gene expression, and BGLR^39^, a β-glucosidase activator, were considered as it.

ACE2 is a transcription factor that enhances the transcriptional activation of *cbh1,* *cbh2,* *egl1,* *egl2,* and *xyn2* in *T.* *reesei* with cellulose induction^34^. However, ACE2 is not involved in sophorose induction. ACE2 binds to the same promoter motif shared with XYR1, and phosphorylation and dimerization are prerequisites for the binding of ACE2 to its target promoter^40^.

In *T.* *reesei*, ACE3 is known to be involved in cellulase production upon induction with lactose^35^. ACE3 interacts with XYR1 to initiate cellulase production^41^. The introduction of multiple gene copies of *ace3* enhances cellulase gene expression and regulates xylanase gene expression^35^. In addition, C-terminal truncated ACE3 can induce high levels of cellulase and hemicellulase expression in non-induced conditions using glucose and further improve expression in induced conditions using lactose or glucose-sopholose mixture^42,43^. It shows improved induction of cellulase both in the presence and absence of an inducer. In addition, the strain overexpressing wild-type XYR1 or mutated XYR1^A824^^V^ and C-terminal truncated ACE3 improved total secreted protein with lactose as the carbon source. However, when glucose was used as the sole carbon source, further overexpression of mutated XYR1^A824V^ in addition to C-terminal truncated ACE3 did not result in further improvement^42^.

In *T.* *reesei*, VIB1 is also essential for cellulase production and acts as an important regulator of cellulase induction^36,44^. A comparison of the Δ*vib1* and Δ*xyr1* transcriptomes using cellulose as a carbon source showed a high frequency of overlap between cellulose-induced genes regulated by VIB1 and XYR1 targets^45^. This suggests that VIB1 partially regulates cellulase gene expression via XYR1^45^.

CRT1 has been considered one of the most important transporters involved in cellulase induction in *T.* *reesei*^37,38^. Its deletion was found to completely abolish cellulase gene expression upon induction with cellulose and lactose^38^. Recently, CRT1 was validated to transport lactose, cellobiose, glucose, and soporose^46^. In addition, when XYR1 is overexpressed under glucose-containing conditions, *crt1* transcription was maintained at a relatively low level^47^.

BGLR, a β-glucosidase activator, is a transcription factor having a Zn(II)_2_-Cys_6_-type DNA-binding domain^39^. One function of BGLR is to upregulate certain β-glucosidase genes^39^. The *bglr* gene deleted mutant showed increased cellulase production during cellobiose growth.

We examined whether mutated XYR1^V821^^F^ and each of the five factors reportedly associated with it affect the regulation of cellulase and hemicellulase production under inducer-free conditions using glucose as the carbon source.

We used a derivative strain of PC-3-7, a high-performance enzyme-producing strain of *T.* *reesei* that is more productive and tends to be alleviated from CCR in glucose^48–50^. *T.* *reesei* is known to secrete a complete set of cellulases when cultured with inducers such as cellulose, cellobiose, and sophorose^5,51^. In contrast, CBH and EG activities of *T.* *reesei* are known to be higher than those of other microorganisms, but its BGL activity is lower than that of cellulase mixtures from other organisms^52^. This results in cellobiose accumulation and subsequent product inhibition of CBH, reducing enzymatic hydrolysis efficiency. To solve this problem, the addition of BGL is effective in improving enzyme performance. Production costs can be lowered by overexpressing the BGL in *T.* *reesei*. The E1AB1 strain has a β-glucosidase gene from *Aspergillus* *aculeatus* (*Aabgl*) in the PC-3-7 strain^52–54^.

We aimed to develop a strain that does not require an inducer for cellulase production using the E1AB1 as a more practical enzyme-producing strain. We attempted to express cellulase by co-expression of the candidate five factors and mutated XYR1^V821F^, which induces high-level xylanase production under non-inducing conditions. In this study, the defect of insufficient cellulase induction was eliminated by constitutive expression of mutated XYR1^V821F^ and ACE3. Thus, we report that inexpensive production of saccharification enzymes can be achieved without cellulase inducers.

## Results

### Effects of the disruption of repressors and the constitutive expression of mutated XYR1^V821F^

In order to combine the expression of mutated XYR1^V821F^ and its candidate cooperative factors, two genomic insertion sites were required to express mutated XYR1^V821F^ and each factor. Therefore, we used the *ace1* and *rce1* gene regions encoding transcriptional repressors as the insertion sites.

**Figure 1:**
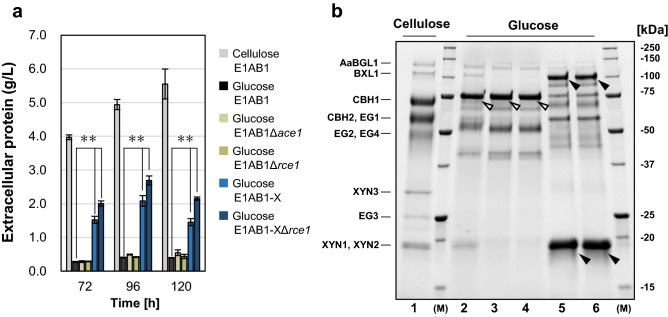
Effects of constitutive expression of V821F mutated XYR1 and disruption of repressors on glucose culture. (**a)** Extracellular protein from the cultivation of samples after 72, 96, and 120 h. *Trichoderma* *reesei* strain E1AB1 was cultivated in shake flasks on an inducing medium containing 3% cellulose, and recombinant strains E1AB1Δ*ace1*, E1AB1Δ*rce1*, E1AB1-X (Δ*ace1*-P*act1*-*xyr1*^V821F^), and E1AB1-XΔ*rce1* were cultivated on a non-inducing medium containing 3% glucose. (**b)** SDS-PAGE analysis of the secreted proteins after 72 h. *T.* *reesei* strain E1AB1 with 3% cellulose (lane 1) and 3% glucose (lane 2) cultures, and recombinant strains E1AB1Δ*ace1* (lane 3), E1AB1Δ*rce1* (lane 4), E1AB1-X (lane 5), and E1AB1-XΔ*rce1* (lane 6) with 3% glucose cultures. Open arrowhead indicates the confirmed in glucose culture (lanes 2–4). Closed arrowheads indicate clearly overexpressed proteins, likely corresponding to the main xylanase (BXL1, XYN1, XYN2, lanes 5 and 6). The gel was cropped in the 15–250 kDa range, and the original gel was shown in the Additional file Fig. S4. Error bars indicate standard deviations. Statistical significance was determined by a two-tailed unpaired Student’s *t*-test. **p < 0.01.

First, disruption of the *ace1* and *rce1* repressor genes was performed to confirm the effect of the deletion. The results showed that protein productivity of E1AB1, Δ*ace1*, and Δ*rce1* strains was reduced to less than 10% under the non-inducing conditions with glucose as the carbon source compared to inducing conditions with cellulose as the carbon source in the parental E1AB1 strain (Fig. 1a). Furthermore, the disruption of *ace1* or *rce1* did not alter the composition of secretory proteins as depicted in Fig. 1b. A high-intensity band indicated by an open arrow was found (Fig. 1b, lanes 2–4) and was identified as a glycoside hydrolase family 55 (GH55) protein (Trire2_121746) by nano LC–MS/MS analysis. This protein was found to be similar to β-1,3-glucanase Lam55A of *Phanerochaete* *chrysosporium* (Additional file Table S1). This indicates that little cellulase production was observed in the three strains with glucose as the sole carbon source.

We selected the *act1* promoter as a constitutive promoter unaffected by carbon source utilization, which showed similar expression levels as pyruvate decarboxylase 1 (Trire2_121534) in RNAseq results obtained under cellulose and glucose-containing culture conditions in the E1AB1 strain^54^. A cassette having XYR1^V821F^ under constitutively active *act1* promoter was introduced at the *ace1* locus, and the strain was named E1AB1-X. The E1AB1-X strain produced 2.08 g/L of extracellular protein under non-inducing conditions. This was a fourfold increase in extracellular protein production compared to the parental strain (Fig. 1a). In addition, differences in band patterns were observed in the SDS-PAGE analysis (Fig. 1b, closed arrowheads). Based on the estimated molecular weights and a previous report^53^, the protein bands likely corresponded to the main xylanases (XYN1, XYN2) and β-xylosidase (BXL1; Fig. 1b, lane 5). However, their composition was different from that of proteins produced by the parental strain E1AB1 in an inducing condition (Fig. 1b, lanes 1 and 5), and the band intensities corresponding to cellulases (CBH1, CBH2, EG1) were also low.

Thus, xylanases could be produced by constitutive expression of the mutated XYR1^V821F^ even under inducer-free conditions; however, cellulases were not fully produced. Further *rce1* gene disruption in the E1AB1-X strain resulted in increased protein productivity (Fig. 1a) but no significant changes in the enzyme composition (Fig. 1b, lane 6). Therefore, the *rce1* locus was used to test the enhancement of cellulase productivity by co-expressing the candidate cooperative factors for cellulase production.

### Co-expression of cellulase transcription-related factors with mutated XYR1^V821F^ in glucose medium to enhance cellulase expression

**Figure 2:**
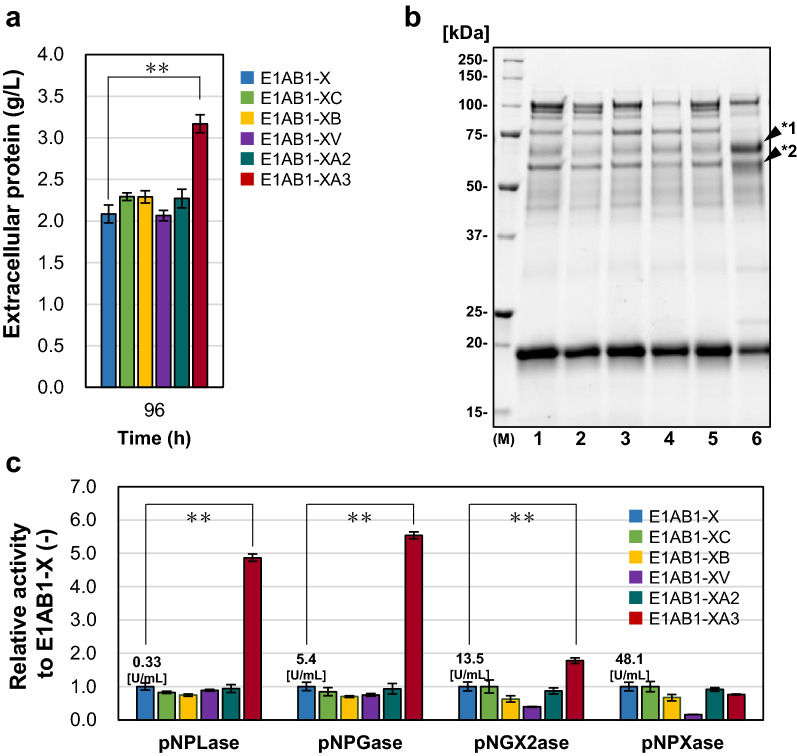
Co-expression of factors involved in cellulase transcription in the E1AB1-X strain. *Trichoderma* *reesei* strain E1AB1-X was co-expressed with various factors that relate to cellulase expression and cultivated in shake flasks on a non-inducing medium containing 3% glucose. (**a)** Extracellular protein from cultivation samples after 96 h. (**b)** SDS-PAGE analysis of the secreted proteins after 72 h in a glucose culture of *T.* *reesei* E1AB1-X (lane 1, Δ*ace1*-P*act1*-*xyr1*^V821F^, as control), E1AB1-XC (lane 2, Δ*ace1*-P*act1*-*xyr1*^V821F^ and Δ*rce1*-P*act1*-*crt1*), E1AB1-XB (lane 3, Δ*ace1*-P*act1*-*xyr1*^V821F^ and Δ*rce1*-P*act1*-*bglr*), E1AB1-XV (lane 4, Δ*ace1*-P*act1*-*xyr1*^V821F^ and Δ*rce1*-P*act1*-*vib1*), E1AB1-XA2 (lane 5, Δ*ace1*-P*act1*-*xyr1*^V821F^ and Δ*rce1*-P*act1*-*ace2*), and E1AB1-XA3 (lane 6, E1AB1-XA3, Δ*ace1*-P*act1*-*xyr1*^V821F^ and Δ*rce1*-P*act1*-*ace3*(PT)). The gel was cropped in the 15–250 kDa range, and the original gel was shown in the Additional file Fig. S5. (**c)** Volumetric enzyme activity of the 72-h culture supernatant. One unit of activity is defined as the amount of enzyme that produced 1 μmol of *p*-nitrophenol per minute per mL of culture supernatant from the substrate at 50 °C. Bar graphs show the activity relative to that of E1AB1-X. Error bars indicate standard deviations. Statistical significance was determined by the two-tailed unpaired Student’s *t*-test. **p < 0.01.

Homologous recombination of *crt1*, *bglr*, *vib1*, *ace2*, and *ace3*, cellulase activators, and factors essential for cellulase expression was done at the *rce1* locus of the E1AB1-X genome, followed by constitutive expression under the *act1* promoter. Constitutive co-expression of CRT1, BGLR, VIB1, or ACE2 in the E1AB1-X strain resulted in no significant changes in the secreted protein concentration (Fig. 2a) and the bands with molecular weights corresponding to cellulases such as CBH1, CBH2 (Fig. 2b, lanes 2–5). In contrast, constitutively ACE3 expressing strain, E1AB1-XA3, showed a 1.5-fold increase in protein production compared to E1AB1-X (Fig. 2a). Enzyme composition analysis revealed a decreased band corresponding to xylanases (XYN1, XYN2, and BXL1) and a few bands with increased intensity, as indicated by closed arrows in E1AB1-XA3 (Fig. 2b, lane 6). These bands, as identified by nano LC–MS/MS analysis, were mainly cellulases (CBH1, CBH2, and EG1), xylanases (BXL1 and XYN4), and small amounts of proteins involved in cellulose degradation (SWO1, CIP2, and EG4) (Additional file Table S2).

To measure the enzyme activity of the culture supernatant of the ACE3 constitutively co-expressing strain, pNPLase was measured for CBH1 activity and pNPGase for BGL activity. The results showed that the E1AB1-XA3 strain was 4.9-fold higher in pNPLase and 5.5-fold higher in pNPGase compared to the E1AB1-X strain. In the E1AB1 strain, the β-glucosidase from *A.* *aculeatus* (*Aabgl1*) is expressed using the *egl1* promoter. The increase in pNPGase activity is suggestive of enhanced transcription of the *egl1* promoter in the E1AB1-XA3 strain. These pNPGase and pNPLase activity values, which indicate cellulase activity, increased about five-fold. While the pNPXase and pNPX2ase activity values, which indicate xylanase activity, changed up to 1.8- and 0.8-folds. The fact that xylanase activity did not improve much, unlike cellulase, suggests that ACE3 contributes to a specific improvement in cellulase expression.

In addition, the band of GH55 observed under non-inducible conditions (Fig. 1b, lanes 2–4 with open arrowhead) disappeared in the supernatant of the ACE3 constitutively expressing strain. Lam55A, which hydrolyzes β-1,3-glucan, could regulate the metabolism of external nutrient sources^55^. Thus, it was inferred that cellulases were induced in the E1AB1-XA3 strain even in the absence of inducers, and the strain resembled the actively induced state for cellulase production. Consequently, we found that the E1AB1-XA3 strain, which combines the constitutive expression of the mutated XYR1^V821F^ and ACE3, makes it possible to produce xylanases and a high yield of cellulases, even under inducer-free conditions.

### Identification of the best combinations of factors required for high protein productivity and cellulase composition under inducer-free conditions

**Figure 3:**
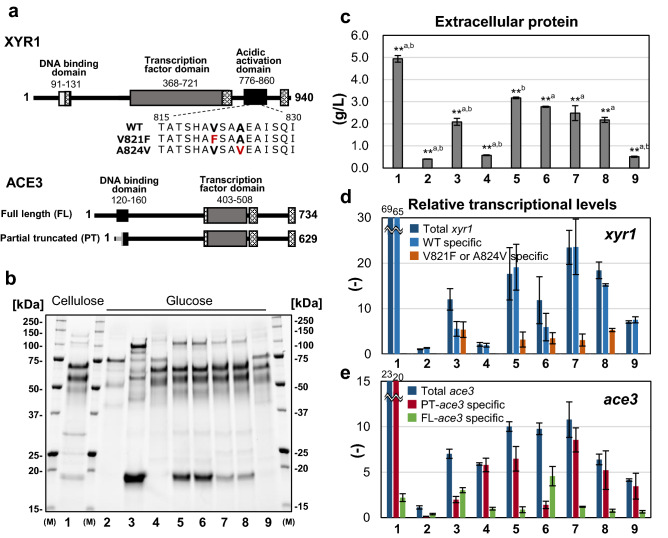
Confirmation of the genetic combinations required for high cellulase production under inducer-free conditions. (**a)** Putative domain of XYR1 and ACE3 used in the study, amino acid mutations in XYR1, and the presumed variant of ACE3. Shaking flask cultivations were performed using 3% cellulose: Lane 1 and 3% glucose: Lane 2–9. The strains used in this figure were Lane 1 and 2: E1AB1, Lane 3: E1AB1-X (Δ*ace1*-P*act1*-*xyr1*^V821F^), Lane 4: E1AB1-A3 (Δ*rce1*-P*act1*-*ace3*(PT)), Lane 5: E1AB1-XA3 (Δ*ace1*-P*act1*-*xyr1*^V821F^ and Δ*rce1*-P*act1*-*ace3*(PT)), Lane 6: E1AB1-XA3fl (Δ*ace1*-P*act1*-*xyr1*^V821F^ and Δ*rce1*-P*act1*-*ace3*(FL)), Lane 7: E1AB1-XA3nhr (P*act1*-*xyr1*^V821F^ and P*act1*-*ace3*(PT) by non-homologous recombination), Lane 8: E1AB1-X824A3nhr (P*act1*-*xyr1*^A824V^ and P*act1*-*ace3*(PT) by non-homologous recombination) and Lane 9: E1AB1-XwtA3nhr (P*act1*-*xyr1*^WT^ and P*act1*-*ace3*(PT) by non-homologous recombination).The carbon sources, strains, and genotypes used in this figure were organized in an Additional file, Table S3. (**b)** SDS-PAGE of secreted proteins after 72 h cultivation (2.5 μg-protein/lane). The gel was cropped in the 15–250 kDa range, and the original gel was shown in the Additional file Fig. S6. (**c)** Extracellular protein concentration at 96 h. Relative transcription levels of *xyr1* (**d**) and *ace3* (**e**). The real-time PCR was performed on the samples taken after 48 h. The transcriptional levels of *pgk1* and E1AB1 strain on glucose culture were measured for reference calculation and data normalization, respectively, and analyzed using the *ΔΔ*Ct method. Error bars indicate standard deviations. Statistical significance was determined by a two-tailed unpaired Student’s *t*-test. **a indicated p < 0.01 for the test with Lane 5 (E1AB1-XA3) and **b indicated p < 0.01 for the test with Lane 7 (E1AB1-XA3nhr).

The E1AB1-XA3 strain had high cellulase productivity even in the absence of inducers, indicating that a combination of ACE3 and mutated XYR1^V821F^ is effective for an increase in cellulase activity (Fig. 2). In addition to V821F mutation, the A824V mutation has been reported previously to be one of the XYR1 mutations that allow expression of xylanase and cellulase expression without inducers^32^. Therefore, to confirm the potentially useful XYR1 mutants, V821F and A824V mutants with a glucose-blind phenotype were evaluated along with the wild-type (Fig. 3a). In contrast, reports have suggested that there is a possibility of multiple transcription start sites in *ace3*^41,43^. The *ace3* of E1AB1-XA3 used in Fig. 2 was derived from the JGI genome database (The ORF sequences (651 or 629 amino acids) presented in http://genome.jgi.doe.gov/Trire2/Trire2.home.html) by reference. However, correct introns and two putative transcription start sites were proposed^41,43^. The estimated complete amino acid sequence was 734 amino acids, and the sequence registered in JGI was a partially truncated N-terminal DNA-binding domain. Therefore, we cloned both putative translation initiation sites from genomic DNA and examined the constitutive expression of partially truncated ACE3 (PT-ACE3) and full-length ACE3 (FL-ACE3) (More detail is provided in the Additional file Fig. S1). In addition, we performed constitutive expression of XYR1 and ACE3 using non-homologous recombination to find whether disruption of the two repressors (*ace1* and *rce1*) was essential. The strains, carbon sources, and genotypes used in this study were listed in the Additional file, Table S3.

In all strains constitutively expressing ACE3, the enzymatic composition of the secreted proteins was mainly cellulase (Fig. 3b, lanes 4, 5, 6, 7, 8, and 9). The total protein production of the E1AB1-A3 strain expressing PT-ACE3 (Fig. 3c, lane 4) and the E1AB1-XwtA3 strain co-expressing ACE3 with wild-type XYR1 (Fig. 3c, lane 9) was 0.57 g/L and 0.51 g/L, respectively. These results indicated that co-expression of ACE3 with the mutated XYR1^V821^^F^ was effective for high productivity. For a mutated XYR1 to be combined need not be V821F-specific but should at least exhibit a glucose-blind phenotype like mutated XYR1^V821F^ or XYR1^A824V^ (Fig. 3b,c, lanes 7 and 8). Furthermore, the co-expression of ACE3 was effective with partial truncated and full-length sequences, both of which had a cellulase-dominated composition and showed higher productivity than a single expression of XYR1^V821F^ (Fig. 3b,c, lane 6). However, the productivity of the E1AB1-XA3fl strain with FL-ACE3 was 14% lower than that of the E1AB1-XA3 strain with PT-ACE3 (Fig. 3c, lane 5). This result was surprising as ACE3 with a partially truncated DNA-binding domain had superior productivity. Expression of mutated XYR1^V821F^ and ACE3 by non-homologous recombination (Fig. 3c, Lane 7, E1AB1-XA3nhr strain) showed the same co-expression effect as above. However, the protein productivity was lower than in the *ace1,* and *rce1* disrupted E1AB1-XA3 strains (Fig. 3c, lane 5).

The transcript levels of *xyr1* and *ace3* were compared in the nine strains listed in Table S3. The total *xyr1* transcript was measured with primer pair at non-mutated regions and was measured for WT, V821F, and A824V specifically by designing a reverse primer at the mutated position. Primers used to measure *ace3* transcripts were designed against common sequences for all *ace3* and in unique sequences to full-length (exon 2) or partial truncated (within intron 2 to exon 3) (Additional file Table S4). Therefore, *xyr1* transcript levels increased 11.5-fold in the E1AB1-X strain, constitutively expressing the mutated XYR1^V821F^ compared to the parental E1AB1 strain in the medium with glucose as a carbon source. The increase was not only in *xyr1*^V821F^ but also *xyr1*^WT^ (native *xyr1*) by 4.2-fold (Fig. 3d, lanes 2 and 3). Furthermore, total *ace3* transcription was increased by 6.2-fold in the E1AB1-X strain, even though *ace3* expression was not directly enhanced (Fig. 3e). This indicates that the expression of native *xyr1* and *ace3* is significantly enhanced directly/indirectly by mutated XYR1^V821F^. Furthermore, in the E1AB1-XA3 strain co-expressing mutated *xyr1*^V821F^ and PT-*ace3*, total *xyr1* and *ace3* transcript levels increased up to 16.8- and 8.9-fold, respectively, compared to the parent strain (Fig. 3d,e, lane 5). In the case of co-expression of *xyr1*^WT^ and PT-*ace3* without utilizing mutated *xyr1* (E1AB1-XwtA3nhr strain), total transcript levels of *xyr1* and *ace3* increased 6.7-fold (Fig. 3d, lane 9) and 3.7-fold (Fig. 3e, lane 9), respectively. However, total secreted protein levels did not increase (Fig. 3c, lane 9). These results suggested that a certain level of total XYR1 or mutated XYR1, such as mutated XYR1^V821F^ or XYR1^A824V^, was required for high cellulase production.

These results also indicated that constitutive expression of the mutated XYR1 exhibiting a glucose-blind phenotype (like V821F, A824V) and ACE3 (full-length and partial truncated) was necessary for high cellulase production in the absence of inducers. Additionally, the partial truncation of the DNA-binding domain of ACE3 and disruptions of *ace1* and *rce1* contributed to high protein productivity, and the genotype of the E1AB1-XA3 strain was found to be the most effective combination in this study.

### Expression and activity analysis of cellulase and xylanase under single or co-expressed XYR1^V821F^ and PT-ACE3

We analyzed the gene expression and activity of cellulase and xylanase in a single expression of XYR1^V821F^ (E1AB1-X strain), a single expression of PT-ACE3 (E1AB1-A3 strain), and co-expression of XYR1^V821F^ and PT-ACE3 (E1AB1-XA3 strain).

**Figure 4:**
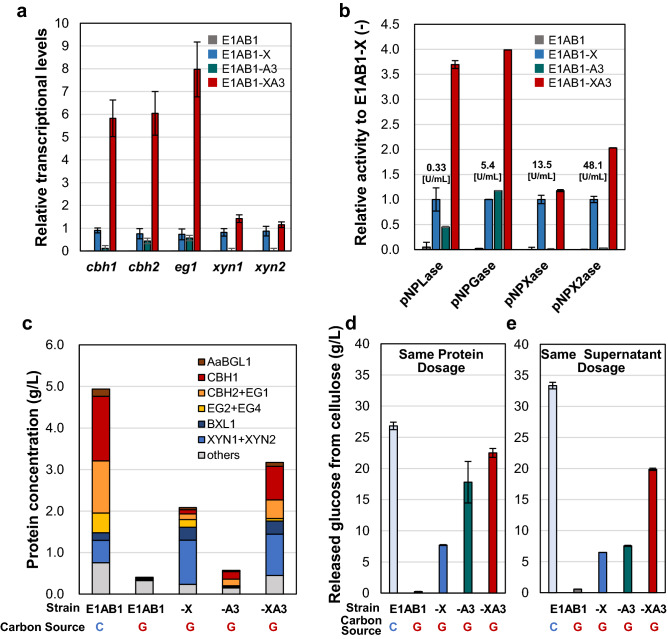
Analysis of gene expression, enzyme activity, composition, and saccharification of microcrystalline cellulose. (**a)** Relative gene expression of the major cellulases and xylanases. The real-time PCR was performed on the 48 h culture sample with glucose as a carbon source. The transcriptional levels of *pgk1* and E1AB1-X strain RNA were measured for reference calculation and data normalization, respectively, and analyzed using the *ΔΔ*Ct method. (**b)** The volumetric activity of each enzyme in the culture supernatant obtained after 72 h of cultivation with glucose as the carbon source. One unit of activity is defined as the amount of enzyme that produced 1 μmol of p-nitrophenol per minute per mL culture of the supernatant from the substrate at 50 °C. Bar graphs show relative activity to E1AB1-X. (**c)** Concentration of each enzyme component was calculated from the enzyme composition and total secreted protein concentration. The compositions were calculated from the band patterns following SDS-PAGE (see Additional file Fig. S2) derived from E1AB1 (culturing using cellulose: C and glucose: G), E1AB1-X (G), E1AB1-A3 (G), and E1AB1-XA3 (G) strains. Protein concentrations were determined from the 96-h value. (**d)** Microcrystalline cellulose saccharification using the same protein dosage (2.0 mg protein/g cellulose). (**e)** Microcrystalline cellulose saccharification using the same volume of culture supernatant (0.87 mL culture supernatant/g cellulose). Error bars indicate standard deviations. Statistical significance was determined by a two-tailed unpaired Student’s *t*-test. **p < 0.01, *p < 0.05.

Owing to the effect of the XYR1^V821F^ mutation, the transcription of major cellulases in the E1AB1-X strain was approximately four orders of magnitude higher than that of the parent strain E1AB1 (Fig. 4a). Furthermore, in the E1AB1-XA3 strain, constitutive expression of PT-ACE3 resulted in an additional 6.4-fold increase in cellulase transcript levels compared to the E1AB1-X strain (Fig. 4a). The increase in expression of the major xylanases*,* *xyn1* and *xyn2* were a maximum of 1.7-fold (Fig. 4a), which was in agreement with their enzymatic activity (Fig. 4b). Consistent with these results, the enzymes produced by the E1AB1-XA3 strain exhibited a similar amount of xylanase and a specific increase in the cellulase (CBH1, CBH2, EG1) components when compared to the E1AB1-X strain (Fig. 4c). The cellulase/xylanase composition of the E1AB1-XA3 strain in the non-induced culture using glucose was similar to that of the induced culture of the parental E1AB1 strain using cellulose (Fig. 4b, Additional file Fig. S2). In the E1AB1-A3 strain, the degrading activities of pNPX2ase and pNPXase were little detected (Fig. 4b), and the bands of BXL1, XYN1, and XYN2 could be little identified (Fig. 3b, Lane No.4). Therefore, constitutive single expression of ACE3 could activate cellulase production but could not fully activate xylanase production (Fig. 3c). Moreover, based on protein concentrations and composition estimated from SDS-PAGE, absolute amounts of CBH1, CBH2, and EG1 were 0.24, 0.34, and 1.25 g/L in the E1AB1-X, -A3, and -XA3 strains, respectively. This confirms a synergistic upregulation of cellulase upon co-expression of mutated XYR1^V821F^ and ACE3 (Fig. 4c).

Saccharification of microcrystalline cellulose was evaluated using enzymes produced under inducing conditions using cellulose or under non-inducing conditions using glucose. In the evaluation using 2.0 mg-enzyme per g-cellulose, the enzyme produced by the parent strain E1AB1 induced with cellulose released the highest amount of glucose at 26.8 g-glucose/L. In contrast, the enzyme produced by strain E1AB1-XA3 was the best in the non-inducing condition with 22.5 g-glucose/L (Fig. 4d). The enzyme from strain E1AB1-A3 released 17.8 g-glucose/L. This result was 2.3-fold higher than that of the enzyme from strain E1AB1-X (Fig. 4d). We inferred that this was perhaps due to the high composition ratio of the cellulase components within the protein. However, as described above, the total amount of secreted enzymes produced by the E1AB1-A3 strain was 0.57 g/L (Fig. 4c), and the absolute amount of the cellulase was 0.34 g/L. In contrast, the enzyme cocktail derived from the E1AB1-X strain contained a protein concentration of 2.09 g/L and was more productive than E1AB1-A3, but it was mainly composed of xylanases; as a result, the absolute amount of cellulases was 0.24 g/L. Therefore, the cellulase component produced by the E1AB1-A3 and E1AB1-X strains was lower than that of the E1AB1-XA3 strain, 1.25 g/L. Therefore, saccharification was performed using 0.87 mL of the same volume of culture supernatant. The results showed that the enzyme from the E1AB1-A3 strain released 6.5 g-glucose/L, and the enzyme from the E1AB1-X strain released 7.5 g-glucose/L, with the two enzymes having comparable performance. In contrast, the enzyme from the E1AB1-XA3 strain released a significantly higher amount, 19.9 g-glucose/L (Fig. 4e). This suggests that the E1AB1-XA3 strain can produce a better enzyme than the existing XYR1^V821F^ overexpression strain under inducer-free condition.

The E1AB1-XA3 strain was found to have two important phenotypes: increased cellulase composition ratio by constitutive expression of ACE3 and high protein productivity under non-inducing conditions by introducing mutated XYR1^V821F^. Thus, we could achieve the most crucial property of the cellulolytic enzyme, which is the increased cellulase production in a non-inducing production system.

## Discussion

The previously reported inducer-free enzyme production system by *T.* *reesei* using mutated XYR1^V821F^ or XYR1^A824V^ mainly produced xylanase, with weak activation of cellulase expression^32,33^. Therefore, we developed a genetic modification strategy to produce both cellulase and xylanase at the same level as in induced conditions. As a result, we could successfully generate large quantities of cellulase and xylanase without inducers. Enhanced cellulase productivity using glucose as the sole carbon source was achieved by constitutive expression of mutated XYR1^V821F^ or XYR1^A824V^ and ACE3, especially ACE3, having a partially truncated DNA-binding domain.

In this report, co-expression of XYR1^V821F^ with cellulase regulation-related factors, CRT1, BGLR, VIB1, or ACE2, did not increase cellulase expression (Fig. 2). Previously, the deletion of these factors was reported to cause loss or reduction of cellulase expression^38–40,45^. These factors were also affected by enhanced expression of XYR1 and ACE3 (for example, *crt1* is regulated by ACE3^38,47^). This suggested that the effect of co-expression of XYR1^V821F^ with these factors may not have occurred because sufficient amounts of these factors were already upregulated by the constitutive expression of mutated XYR1^V821F^ and subsequent upregulation of ACE3 (Fig. 3e, Lane No. 3). In contrast, the effect of ACE3 co-expression was observed to contribute to the improved cellulase production (Fig. 2), suggesting that it is necessary to enhance the expression of ACE3 beyond the upregulation by mutated XYR1^V821F^. It was previously reported that ACE4 directly binds to the *ace3* promoter and regulates ACE3 expression to promote cellulase production^56^. Possibly ACE3 is regulated not only by XYR1 but also by other factors, including ACE4, which suggests that enhanced expression of ACE3 by factors other than XYR1 may be the key to the cellulase expression. In addition to the regulation of main cellulase/hemicellulase expression, it is also possible that minor enzymes (not under the control of XYR1 and ACE3) were not expressed, and further comprehensive expression profile analysis of various regulators and glycoside hydrolase families requires future intervention.

XYR1 has been studied as one of the key regulators of cellulase and xylanase production, but its detailed regulatory mechanism remains unclear. In our study, co-expression of wild-type XYR1 and PT-ACE3 (E1AB1-XwtAnhr strain) did not show an increase in cellulase productivity compared to the constitutively expressing PT-ACE3 (E1AB1-A3 strain) (Fig. 3). Furthermore, in an industrial strain derived from Rut-C30, cellulase production did not increase in the absence of cellulase inducers upon replacing the native *xyr1* promoter with constitutive promoters, such as promoters from phosphoglycerate kinase, histone 3, and basic-leucine zipper transcription factor genes^12^. In contrast, overexpression of wild-type XYR1 using the copper-repressible promoter P*tcu1* in the *T.* *reesei* QM9414 strain resulted in high cellulase production in glucose-enriched cultures^57^. Although the *act1* promoter was selected as the constitutively expressed promoter in this study, it might have been insufficient to enhance the expression level of wild-type *xyr1*, and possibly the cellulase could be induced by overexpression of wild-type XYR1. In contrast, expression of mutated *xyr1*^V821F^ using the same *act1* promoter led to remarkable xylanase production in non-inducing conditions (Fig. 1). It is also suggested that post-translational modification of XYR1 may be required for complete activation of cellulase and hemicellulase gene transcription^12^. Mutations in the acidic activation domain of XYR1, such as A824V or V821F, could mimic post-translational modifications and activate at least xylanase expression at lower levels than wild-type XYR1. Therefore, it was suggested that the expression profiles of cellulase/hemicellulase are different even using the same promoter strength in wild-type and mutated XYR1, and further investigation is needed to determine if the expression level of mutated XYR1^V821F^ was optimal in this study and the effect of promoter strength to driving the wild-type/mutated XYR1.

Another modification factor, ACE3, has been shown to play an important role in cellulase regulation, but its functional analysis remains unclear. ACE3 in *T.* *reesei* NG14 and derived RUT-C30 and RL-P37 strains have an 11-amino acid truncation at the C-terminus, resulting in higher cellulase productivity^42^. Furthermore, Luo et al*.* reported high cellulase production even under inducer-free conditions by overexpression of ACE3 with C-terminal truncation of 7–17 amino acids, using the RL-P37 strain as the parent strain^43^. In this reported system, none of the *ace3* had a complete wild-type C-terminus^43^. In contrast, in this study, in the E1AB1 strain derived from the PC-3–7 strain, *ace3* in the genome had a complete C-terminus, and *ace3* with further enhanced expression had no C-terminal mutation. However, high cellulase production was achieved under non-inducing conditions by co-expression with mutated XYR1^V821F^ (Fig. 3a). Luo et al*.* suggested that the C-terminal 17 amino acids of ACE3 may be either the repressor domain itself or part of a domain that interacts with the repressor^43^. Our results indicated that the C-terminal mutation of ACE3 is not essential for inducer-free production of cellulase and that its interaction with mutated XYR1^V821F^ may directly or indirectly release its inhibitory function at the C-terminus of ACE3.

In addition, Luo et al*.* reported that a Zn(II)_2_Cys_6_-type DNA-binding domain with all six cysteines at the N-terminus and specific mutation at the C-terminus were required for inducer-free cellulase production^43^. In contrast, in the present study, the expression of FL-ACE3 with a complete N-terminal DNA-binding domain enhanced cellulase productivity. However, surprisingly, PT-ACE3 with an incomplete DNA-binding domain was more productive (Fig. 3). ACE3 was proposed to form homodimers with ACE3 and heterodimers with XYR1^41^, and constitutively express PT-ACE3 which possibly interact with native-ACE3 (having complete DNA-binding domain), PT-ACE3, native-XYR1, or mutated XYR1^V821F^. In addition, the transcript levels of FL-*ace3* were not significantly increased under induced conditions using cellulose, and transcripts mainly corresponding to PT-*ace3* increased (Fig. 3e, lane 1), supporting the previous reports^43^. Therefore, ACE3 with DNA binding ability is required for cellulase expression, but not necessarily to increase the expression level. It was considered that ACE3 corresponding to PT-ACE3 regulates cellulase expression through dimer formation and interactions with other factors also during cellulase production with inducers. The DNA-binding domain of ACE3 is classified as a Gal4-like Zn(II)_2_Cys_6_ binuclear cluster; Gal4 is known to bind DNA as a homodimer^58^. Therefore, it was speculated that homodimers with PT-ACE3 (with PT-ACE3 or native FL-ACE3), in which a part of the DNA-binding domain was truncated, lose DNA-binding ability. However, the results of the co-expression and regions of increased transcription during induced production in Fig, 3e suggested that the presence of PT-ACE3 positively regulates cellulase production. Therefore, DNA binding of ACE3 may also play a role in inhibition, depending on the state. It was also thought that heteromerization with XYR1, which can bind DNA, could activate cellulase production, but further investigation into the function of ACE3 with partial truncation of the DNA-binding domain would be needed.

The *ace1*^25^ and *rce1*^26^ loci used to insert *xyr1*^*V821F*^ and other cellulase regulation-related factors were known repressors. In this study, their disruption was not essential and did not contribute to improved cellulase composition, although the total secreted protein concentration was increased (Fig. 3b,c). The enzyme productivity of the E1AB1-XA3 strain using glucose was improved compared to the E1AB1-X and E1AB1-A3 strains but slightly inferior to the parent E1AB1 strain using cellulose (Figs. 1a, 3c, 4c). This seemed to be largely dependent on the property of cellulose as a carbon source and its inducibility. Glucose is extremely easy to assimilate and is consumed rapidly; therefore, it was depleted after 48 h in this study (Additional file Fig. S3). In contrast, cellulose is not easily assimilated and degrades slowly, releasing cellobiose and glucose over a long time, and it is considered less likely to induce CCR. The E1AB1 strain used in this study is an industrial strain that has undergone repeated mutation breeding, and the *cre1* mutation^50^ and α-tubulin (*tubB*) disruption^54^ have eliminated various suppressive regulations, including CCR. However, even in this strain, enzyme production was slight, while glucose concentrations were high (Additional file Fig. S3). Luo et al*.*^43^ also mentioned that (hemi) cellulase production only occurs after the glucose has been depleted below a certain threshold, and the CCR was not fully overcome. In the present state, the practices to avoid CCR (for example, fed-batch culture^59,60^) are necessary to lead to high cellulase productivity equivalent to cultures using cellulose, and constitutive expression of activators such as XYR1 and ACE3 alone cannot overcome the CCR. Therefore, further investigation of the repression mechanisms is needed in addition to modification of these activators.

Due to its high production ability, *T.* *reesei* is a promising host for heterologous protein production. However, using the conventional cellulose-based enzyme production system is difficult for heterologous protein production^61,62^. If the cellulase genes are replaced with genes for the proteins of interest, then cellulose cannot be decomposed, and the inducer is not released, diminishing the protein production ability. Contrarily, if cellulase is not disrupted, large amounts of native cellulases will be contaminated^62^. Previous reports showed heterologous protein production using *xyn1* and *xyn2* promoters with *xyr1*^*A824V*^^63^. However, the results of this study indicate that it has become possible to use extremely strong cellulase promoters, such as *cbh1* and *cbh2*, for heterologous protein production without the co-production of cellulase. Such strong cellulase promoters are expected to be great productivity tools that can be used to express saccharifying enzymes for bioethanol production and various valuable proteins.

## Methods

### Strains and propagation

The *T.* *reesei* strains used in this investigation are listed in Table 1. *T.* *reesei* strains E1AB1^53^ and E1AB1Δ*pyr4* (uracil auxotroph) were kindly provided by Prof. W. Ogasawara (Nagaoka University of Technology). Strains were maintained on potato dextrose agar (PDA; Difco Laboratories, Detroit, MI) plates.

**Table 1:** *Trichoderma* *reesei* strains used in this study.

Strain	Genotypes	References
PC-3-7	–	^48^
E1AB1	*amdS*^+^, P*egl1*-*Aabg1*	^53^
E1AB1Δ*ace1*	*amdS*^+^, P*egl1*-*Aabg1*, Δ*ace1*	This study
E1AB1Δr*ce1*	*amdS*^+^, P*egl1*-*Aabg1*, Δr*ce1*	This study
E1AB1-X	*amdS*^+^, P*egl1*-*Aabg1*, Δ*ace1*::P*act1*-*xyr1* (V821F)	This study
E1AB1-XΔ*rce1*	*amdS*^+^, P*egl1*-*Aabg1*, Δ*ace1*::P*act1*-*xyr1* (V821F), Δ*rce1*	This study
E1AB1-XC	*amdS*^+^, P*egl1*-*Aabg1*, Δ*ace1*::P*act1*-*xyr1* (V821F), Δ*rce1*::P*act1*-*crt1*	This study
E1AB1-XB	*amdS*^+^, P*egl1*-*Aabg1*, Δ*ace1*::P*act1*-*xyr1* (V821F), Δ*rce1*::P*act1*-*bglr*	This study
E1AB1-XV	*amdS*^+^, P*egl1*-*Aabg1*, Δ*ace1*::P*act1*-*xyr1* (V821F), Δ*rce1*::P*act1*-*vib1*	This study
E1AB1-XA2	*amdS*^+^, P*egl1*-*Aabg1*, Δ*ace1*::P*act1*-*xyr1* (V821F), Δ*rce1*::P*act1*-*ace2*	This study
E1AB1-XA3	*amdS*^+^, P*egl1*-*Aabg1*, Δ*ace1*::P*act1*-*xyr1* (V821F), Δ*rce1*::P*act1*-*ace3* (PT)	This study
E1AB1-XA3fl	*amdS*^+^, P*egl1*-*Aabg1*, Δ*ace1*::P*act1*-*xyr1* (V821F), Δ*rce1*::P*act1*-*ace3* (FL)	This study
E1AB1-A3	*amdS*^+^, P*egl1*-*Aabg1*, Δ*rce1*::P*act1*-*ace3* (PT)	This study
E1AB1-XA3nhr	*amdS*^+^, P*egl1*-*Aabg1*, P*act1*-*xyr1* (V821F), P*act1*-*ace3* (PT)	This study
E1AB1-X824A3nhr	*amdS*^+^, P*egl1*-*Aabg1*, P*act1*-*xyr1* (A824V), P*act1*-*ace3* (PT)	This study
E1AB1-XwtA3nhr	*amdS*^+^, P*egl1*-*Aabg1*, P*act1*-*xyr1* (WT), P*act1*-*ace3* (PT)	This study

### Shake flask cultivation

For preculture enzyme production, 4 × 10^5^ spores of each strain were inoculated into 2 mL of basal medium^48^ containing 1% (w/v) glucose in a 10-mL culture tube. Spores were counted using a Thoma hemocytometer (Sunlead Glass Corp.). The basal medium comprised 0.14% (w/v) (NH_4_)_2_SO_4_, 0.2% (w/v) KH_2_PO_4_, 0.03% (w/v) CaCl_2_·2H_2_O, 0.03% (w/v) MgSO_4_ 7H_2_O, 0.1% (w/v) polypeptone, 0.05% (w/v) yeast extract, 0.1% (w/v) Tween 80, and 0.1% (w/v) trace element solution in 50 mM Na-tartrate buffer (pH 4.0). The trace element solution contained 6 mg H_3_BO_3_, 26 mg (NH_4_)_6_Mo_7_O_24_·4H_2_O, 100 mg FeCl_3_·6H_2_O, 40 mg CuSO_4_·5H_2_O, 8 mg MnCl_2_·4H_2_O, and 200 mg ZnCl_2_ in 100 mL of distilled water. Preculturing was carried out by shaking at 220 rpm, at 28 °C for 2 days. For each of the main cultures, 500 μL of the preculture was inoculated into 50 mL of basal medium containing 3% (w/v) powdered cellulose (KC FLOCK W-400G, Nippon Paper Industries) or glucose and 1.28% (w/v) diammonium hydrogen citrate in a 500 mL Erlenmeyer flask. The main culture was shaken at 220 rpm at 28 °C for 3–5 days. For sampling, cells were removed from the culture broth by centrifugation at 16,000*g* for 5 min, and the supernatant was filtered through a 0.20 μm cellulose acetate membrane filter (13CP020AN; Advantec, Toyo Roshi Kaisha). All experiments were carried out in triplicate.

### Molecular cloning and construction of the expression cassettes

The genes of interest were amplified from the genomic DNA of *T.* *reesei* strain PC-3–7, and a vector fragment was amplified by inverse polymerase chain reaction (PCR) using pUC118 (Takara Bio) as the template. The amplified fragments, designed using primers that added a SwaI cleavage site, were ligated using an In-Fusion HD Cloning Kit (Clontech) according to the manufacturer’s protocol. *Escherichia* *coli* DH5a was used as a cloning host, and a NucleoSpin® Plasmid miniprep kit (Takara Bio) was used to purify the plasmid DNA. More details on the cloned gene and primers are provided in the Additional file, Table S5. The vector fragment pUC-K017 was generated by inverse PCR; it was derived from the ligated *ace1* inserted plasmid (pUC-K003) and *pyr4* marker cassette (pUC-K016). An inverse PCR fragment of the *ace1* disruption construct (pUC-K017) and fragments of the *act1* promoter, *xyr1*, and *cbh1* terminator was ligated to generate the plasmid ΔAce1-P*act1*-*xyr1*-*pyr4* (pUC-K019). Using this plasmid as a template, inverse PCR was performed to mutate valine to phenylalanine at position 821 to obtain ΔAce1-P*act1*-*xyr1*^V821F^-*pyr4* (pUC-K020) and to mutate alanine to valine at position 824 to obtain ΔAce1-P*act1*-*xyr1*^A824V^-*pyr4* (pUC-K021). In the same manner, an inverse PCR fragment of the *rce1* disruption construct (pUC-K018) and fragments of the *act1* promoter, ORFs (*crt1*, *bglr*, *vib1*, *ace2*, PT-*ace3*, FL-*ace3*), and the *cbh1* terminator were ligated to generate the plasmids pUC-K022, pUC-K023, pUC-K024, pUC-K025, pUC-K026, and pUC-K027, respectively. For PT-*ace3* and FL-*ace3*, pUC-K010 was used as a template to design a forward primer based on the two types of putative initiation codons. More details on vector construction and the primers used are provided in the Additional file, Table S6.

### Transformation of *T. reesei*

Plasmids were linearized with SwaI prior to the transformation of *T.* *reesei*, or the PCR product was transformed using a modified protoplast-PEG method^64^, in which 20 mg/mL of Yatalase (Takara Bio) was used as the protoplasting enzyme instead of Novozyme 234 (Novozymes Bagsværd, Denmark). The transformed protoplasts were plated on minimal transformation medium [2.0% (w/v) glucose, 18.27% (w/v) sorbitol, 0.5% (w/v) (NH_4_)_2_SO_4_, 0.2% (w/v) CaCl_2_, 0.06% (w/v) MgSO_4_, 0.21% (w/v) CsCl, and 0.1% (w/v) trace element solution in 100 mM KH_2_PO_4_ buffer (pH 5.5)] for the *pyr4* marker. The trace element solution contained 500 mg FeSO_4_·7H_2_O, 200 mg CoCl_2_, 160 mg MnSO_4_·H_2_O, and 140 mg ZnSO_4_·7H_2_O in 100 mL of distilled water. After two weeks of incubation at 30 °C, candidate transformants were streaked twice on selective plates (each minimal transformation medium without sorbitol) for several days at 30 °C for single-colony isolation. Single colonies were then transferred to PDA plates for one week at 30 °C to allow for the formation of conidia. According to the manufacturer’s protocol, one transformant was confirmed by colony PCR using KOD One (Toyobo). To transform the resulting transformants again using the PDA medium containing 0.2% (w/v) 5-fluoroorotic acid (5-FOA) monohydrate, a strain that acquired 5-FOA resistance again (*pyr4* pop-out through homologous recombination) was selected.

### Culture supernatant and cell biomass analysis

According to the manufacturer’s protocol, protein concentration was determined using the Bradford protein assay (Bio-Rad) with bovine gamma globulin as the standard. The glucose concentration in the supernatant was quantified using the Glucose CII Test Wako Kit (Wako Chemicals). Specifically, 150 μL of the reaction solution was added to 1 μL of the diluted supernatants in a 96-well plate, mixed well, and incubated at room temperature for 15 min. The absorbance of all samples and standards was measured at 505 nm using a microplate reader (Molecular Devices).

### Real-time quantitative PCR (RT-qPCR) analysis

The cell pellet at 48 h, collected by centrifugation, was lightly dewatered and frozen in liquid nitrogen. A metal cone was placed in the frozen sample prior to crushing with a Multi-Beads Shocker (Yasui Kikai Corp.) at 1700 rpm for 10 s, and RNA extraction was subsequently performed using the RNeasy Mini Kit (Qiagen), according to the manufacturer’s protocol. gDNA digestion and cDNA synthesis were performed using ezDNase™ Enzyme (Invitrogen) and SuperScript™ IV VILO™ Master Mix (Invitrogen), respectively. RT-qPCR experiments were performed with Brilliant III Ultra-Fast SYBR Green QPCR Master Mixes (Agilent), and the transcriptional levels were evaluated using the ΔΔCt method using *pgk1* gene as a normalizer and E1AB1 (Fig. 3d,e) or E1AB1-X (Fig. 4a) strain as calibrator. All samples were analyzed in at least three independent biological experiments. The primers used for real-time PCR were designed using Primer3 (https://bioinfo.ut.ee/primer3-0.4.0/) and are listed in the Additional file Table S4.

### Enzyme composition analysis

SDS-PAGE was carried out using Any kD Mini-PROTEAN TGX Precast Protein Gels (Bio-Rad) for 35 min at 200 V. The gel was activated for 5 min and imaged using the ChemiDoc MP imaging system (Bio-Rad). Precision Plus Protein Unstained Standard (5 μL; Bio-Rad) was used as a molecular mass marker. Unless otherwise noted, 2.5 μg of protein was loaded in each well. The gels were cropped in the 15–250 kDa range because no clear bands were detected under 15 kDa (the original gel of Figs. 1b, 2b, and 3b were presented in Fig. S4, S5, and S6 see Additional file). The molecular weight of the protein bands was estimated using Image Lab software (BiFio-Rad), and the protein bands were annotated using the positions corresponding to previously reported cellulases and xylanases^53^. Protein identification using nano LC–MS/MS systems was performed in Japan Proteomics Co. LTD.

### Enzyme activity analysis

The enzymatic activities of cellobiohydrolase, β-glucosidase, xylanase, and β-xylosidase were measured using the substrates *p*-nitrophenyl-β-d-lactoside (pNPL), *p*-nitrophenyl-β-d-glucopyranoside (pNPG), *p*-nitrophenyl-β-xylobioside (pNPX2), and *p*-nitrophenyl-β-d-xylopyranoside (pNPX), respectively. The reactions were carried out in 50 mM sodium acetate at pH 5.0 and 50 °C for 10 min and terminated by adding one volume of 1 M Na_2_CO_3_. The released *p-*nitrophenol was quantified by measuring the absorbance at 420 nm. One unit of activity is defined as the amount of enzyme that produced 1 μmol of *p*-nitrophenol per minute at 50 °C. Cellulose saccharification was performed on a 1-mL scale in 9-mL glass screw-top bottles at a loading of 5% (w/v) microcrystalline cellulose (Avicel^®^ PH-101, Sigma-Aldrich) in 100 mM sodium acetate buffer pH 5.0, using an enzyme loading of 2.0 mg protein/g cellulose. The protein concentration was determined as mentioned above. The reaction was performed at 50 °C under shaking at 150 rpm for 72 h. Samples obtained following saccharification were filtered (0.2 μm), and the glucose concentration was measured using an enzymatic assay and a Multifunction Biosensor BF-7 (Oji Scientific Instruments), according to the manufacturer’s protocol.

### Statistical analysis

All experiments were performed with at least three independent samples. Error bars indicate the standard deviation (SD) of the mean of triplicates. Statistical significance was determined by the two-tailed unpaired Student’s t-test. Within each set of experiments, *p* < 0.05 was considered significant.

## Supplementary Information

Supplementary Information.

## Data Availability

The protein and nucleotide sequences used in this study can be referenced from Uniprot under the following accession IDs: ACE1_G0RCC6, RCE1_G0RBV8, XYR1_G0RLE8, CRT1_G0RGH7, BGLR_G0RVU2, VIB1_G0R8Z5, ACE2_G0RKV9, ACE3 (partially truncated)_G0RIA0, ACE3 (full-length)_A0A5C1J077.

## Abbreviations

BGL: β-Glucosidase
BXL: β-Xylosidase
CBH: Cellobiohydrolase
CCR: Carbon catabolite repression
EG: Endoglucanase
PCR: Polymerase chain reaction
pNPG: *p*-Nitrophenyl-β-d-glucopyranoside
pNPL: *p*-Nitrophenyl-β-d-lactoside
pNPX: *p*-Nitrophenyl-β-d-xylopyranoside
pNPX2: *p*-Nitrophenyl-β-xylobiosid
XYN: Xylanase
