# Supplementary material for: Inducer-free cellulase production system based on the constitutive expression of mutated XYR1 and ACE3 in the industrial fungus Trichoderma reesei
Source: Sci Rep. 2022 Nov 14;12:19445. doi: 10.1038/s41598-022-23815-4 (PMC9663580; doi:10.1038/s41598-022-23815-4)
Supplement: Supplementary file 1 — Supplementary Information. [file 41598_2022_23815_MOESM1_ESM.pdf]

Table S1 Identification of proteins shown as open arrowheads in Fig. 1b using nanoLC/MS-MS.

	Protein		MW	Score	Peptide	Coverage	Accession	*Note*		
1	Glycoside hydrolase family 55		83,086	669	33	30	G0RJ25	*XP_006965432.1*		
2	Glycoside hydrolase family 55		79,800	275	11	22	G0R927	*XP_006961802.1*	TRIREDRAFT_121746_EGR48699	(GH55)
3	Glycoside hydrolase family 55		79,376	76	6	8	G0RX78	*XP_006969845.1*		
4	Alpha-galactosidase 3	AGL1	68,455	40	3	6	Q92451	*sp|Q92451.1| AGAL3_HYPJE*	TRIREDRAFT_72704_EGR44740	AGL1

Table S2 Identification of proteins shown as closed arrowheads in Fig. 2b using nanoLC/MS-MS.

*1	Protein		MW	Score	Peptide	Coverage	Accession	*Note**		
1	Exoglucanase 1	CBH1	54,073	442	20	25	P62694	*pdb|1CEL|A*	TRIREDRAFT_123989_EGR44817	CBH1
2	Non-Catalytic module family expansin	SWO1	51,524	255	11	33	G0RVK3	*XP_006969225.1*	TRIREDRAFT_123992_EGR44818	SWO1
3	Xylan 1,4-beta-xylosidase	BXL1	87,191	140	8	13	G0RG23	*XP_006964075.1*	TRIREDRAFT_121127_EGR49702	BXL1
4	Alpha-glucuronidase	GLR1	93,424	31	2	2	Q99024	*sp|Q99024.1| AGUA_HYPJE*	TRIREDRAFT_72526_EGR44925	GLR1
*2	Protein		MW	Score	Peptide	Coverage	Accession	*Note*		
1	Exoglucanase 2	CBH2	49,653	409	19	17	A0A024SH76	*sp|A0A024SH76.1| GUX2_HYPJR*	TRIREDRAFT_72567_EGR51017	CBH2
2	Exoglucanase 1	CBH1	54,111	240	13	17	A0A024RXP8	*sp|A0A024RXP8.1| GUX1_HYPJR*	TRIREDRAFT_123989_EGR44817	CBH1
3	Endoglucanase EG-1	EG1	48,208	139	4	14	A0A024SNB7	*sp|A0A024SNB7.1| GUN1_HYPJR*	TRIREDRAFT_122081_EGR48251	EG1
4	Glycoside hydrolase family 5	XYN4	52,845	439	15	29	G0RVK4	*XP_006969226.1*	TRIREDRAFT_111849_EGR44819	XYN4
5	4-O-methyl-glucuronoyl methylesterase	CIP2	48,296	56	1	2	G0RV93	*sp|G0RV93.1| GCE_HYPJQ*	TRIREDRAFT_123940_EGR44948	CIP2
6	Endoglucanase-4	EG4	35,511	46	2	5	O14405	*sp|O14405.1| GUN4_HYPJE*	TRIREDRAFT_73643_EGR52697	EG4
7	Cell wall protein		41,197	37	1	2	G0RTC0	*XP_006968530.1*	TRIREDRAFT_68067_EGR45612	

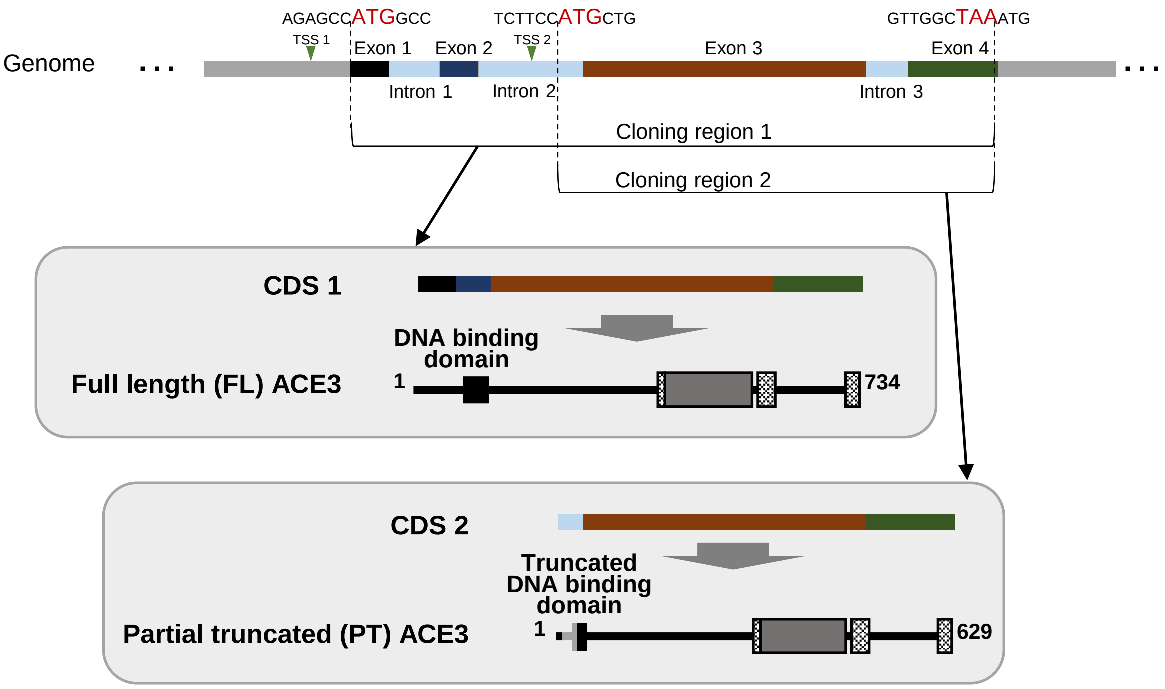

Figure S1 Genomic sequence of *ace3*, cloning region, and putative FL-ACE3 and PL-ACE3 sequences translated from those sequences. According to previous reports[40, 42], two different transcripts were identified at the *ace3*, one transcript was mapped to initiate at a position ~ 78 bp upstream of the first start codon (TSS 1), and the other was mapped to start within intron 2 at ~ 148 bp upstream of the putative second start codon (TSS2). The presence of the 5′ UTR in these two transcripts suggested that they do not originate from alternative splicing, but from two different transcription start sites[42]. We cloned regions 1 and 2 and used the *act1* promoter to constitutively express these ACE3.

Table S3 Strains, carbon sources, and genotypes used in Fig. 3.

Table S3 Strains, carbon sources, and genotypes used in Fig. 3

**Lane No.**				**1**	**2**	**3**	**4**	**5**	**6**	**7**	**8**	**9**
**Strain**				E1AB1	E1AB1	EIAB1 -X	EIAB1 -A3	EIAB1 -XA3	EIAB1 -XA3fl	EIAB1 -XA3nhr	EIAB1 -X824A3nhr	EIAB1 -XwtA3nhr
**Carbon Source**				Cel	Glc	Glc	Glc	Glc	Glc	Glc	Glc	Glc
**Genotypes**		**promoter**	**CDS**									
	xyr1	native	*xyr1* ^{WT}	+	+	+	+	+	+	+	+	+
		act1	*xyr1* ^{WT}	-	-	-	-	-	-	-	-	+
		act1	*xyr1* ^{V821F}	-	-	+	-	+	+	+	-	-
		act1	*xyr1* ^{A824V}	-	-	-	-	-	-	-	+	-
	ace3	native	*ace3*	+	+	+	+	+	+	+	+	+
		act1	*ace3* (PT)	-	-	-	+	+	-	+	+	+
		act1	*ace3* (FL)	-	-	-	-	-	+	-	-	-
	ace1			-	-	deletion	-	deletion	deletion	-	-	-
	rce1			-	-	-	deletion	deletion	deletion	-	-	-

Table S4 Primer pairs for Real-Time PCR.

Gene	Forward Primer(5'→3')	Reverse Primer(5'→3')
Total *xyr1*	GATGAGCACGACTTGAACAGAAA	ACGATGTAAACGAGCCACCA
xyr1 ^{WT} specific	CATGTCCTCCACATCCTCCTT	ATAGCTTCGGCAGCCGATAC
xyr1 ^{V821F} specific	CATGTCCTCCACATCCTCCTT	ATAGCTTCGGCAGCCGAGAA
xyr1 ^{A824V} specific	CATGTCCTCCACATCCTCCTT	GAGAATCTGGCTAATAGCTTCGAC
Total *ace3*	AAGGACATTTTCCCCGAAGG	GGAGGCAGTTGGAGTGGAAG
PT-*ace3* specific	ACCACTGACCAATGCTCTTCC	TCGCTCTTCTTCCTCGCTTT
FL-*ace3* specific	ACCACCTCGCCCGAGTCT	GGGATGGTGGTGGAACGAG
cbh1	ACGAGTTCTCTTTCGATGTTGATG	GCGGTGTTGGTGGGATACTT
cbh2	GTCGCAAAGGTTCCCTCTTTT	CGGCAAGTCATACACCACAAA
eg1	GCTCGTGTTCAGCATTTGGA	TGCGTGTTGGGGTTGTTG
xyn1	AGCATCAACTACGACCAAAACTACC	ACCAACGCCCACAACAAAG
xyn2	TCAACACGGCGAACCACTT	AACCCTCCACGGCAACAA
bxl1	TTGAGTTTGAGTTGGTGGGAGA	AGGTGTAGCATCCTTGATCTGTTG
pgk1	TCTGGGCGACATCTACAT	GTAGTCGAGCTCCTTCTTCA

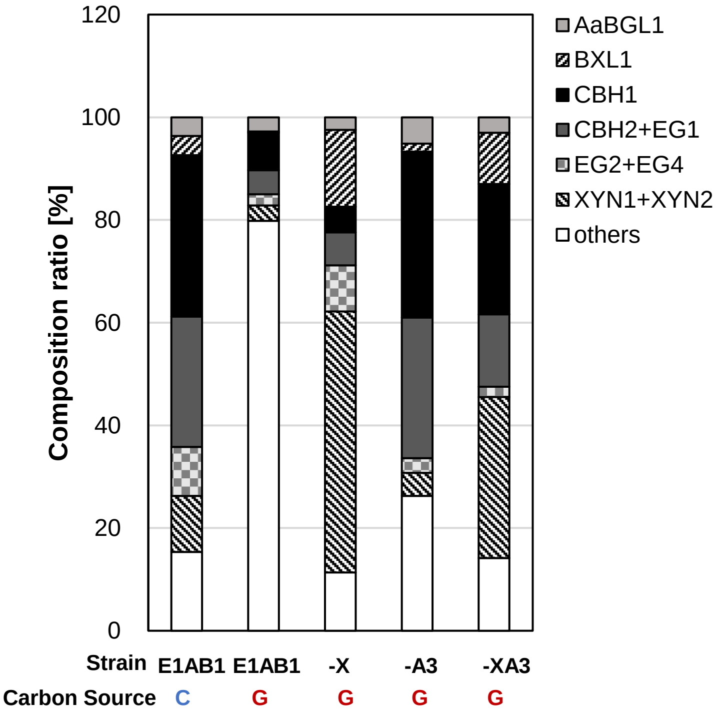

Figure S2 Calculated enzyme compositions of the secreted proteins at 72 h using cellulose as a carbon source (C) of *T. reesei* E1AB1,and using glucose as a carbon source (G) of *T. reesei* strains on E1AB1 , E1AB1-X, E1AB1-A3, E1AB1-XA3.

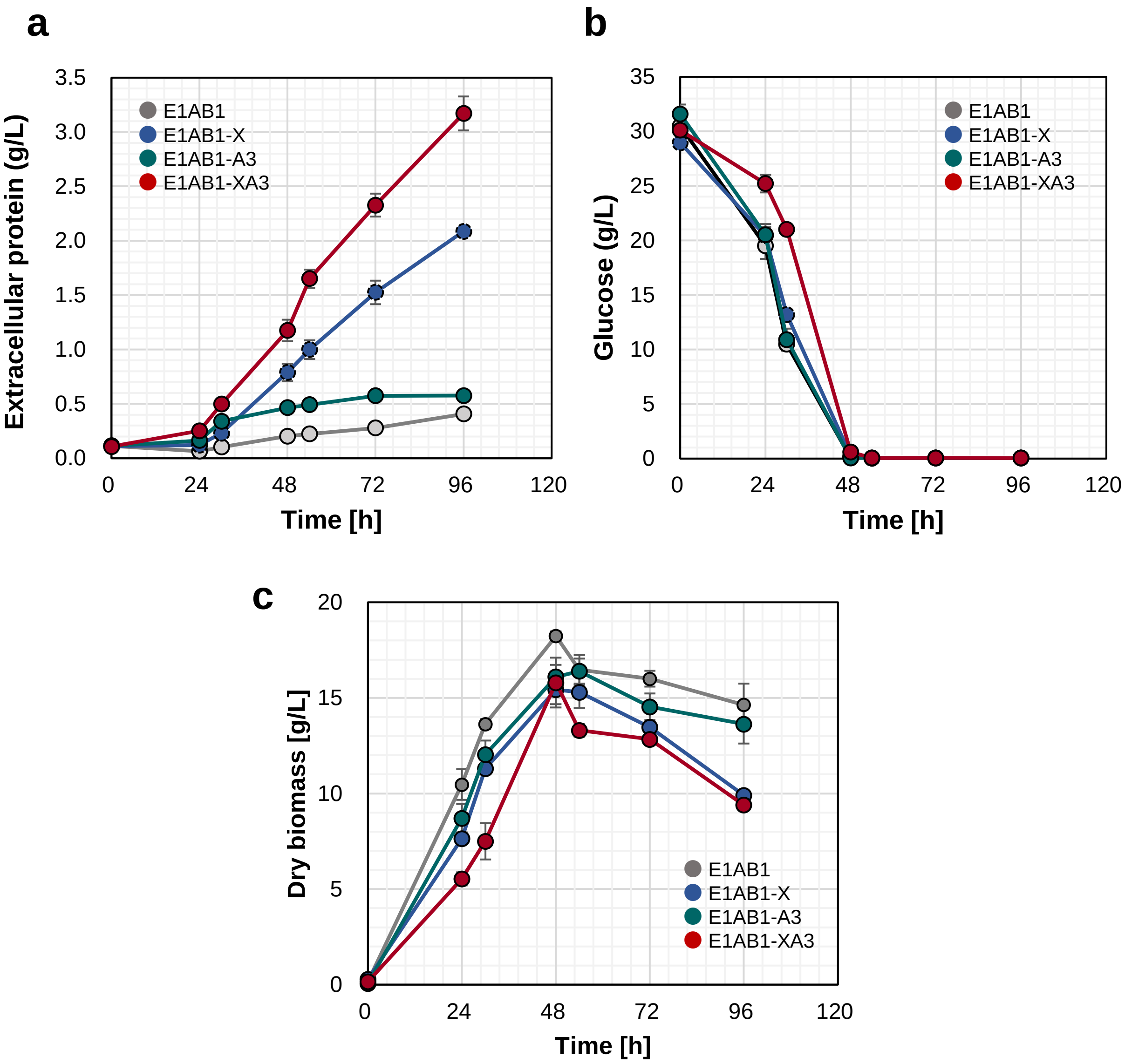

Figure S3 Culture behavior of inducer-free protein production. E1AB1, E1AB1-X, E1AB1-A3, and E1AB1-XA3 strains were cultivated in shake flasks on a non-inducing medium comprising 3% glucose and sampled after 0, 24, 30, 48, 54, 72, and 96 h. **a** Extracellular proteins, **b** Glucose concentration in the supernatant, **c** dry biomass. Error bars indicate standard deviations.

Table S5 Primer pairs for gene cloning.

Plasmid		fragment	Forward Primer	Reverse Primer	Templete
pUC-K001	pUC-118		-	-	-
pUC-K002	pUC-act1	1	CTAGAGTATTTAAATACGGGGTACGCGTGAAATTG	TGCAGGTATTTAAATTGTGACTGATTAATGTATGA	Genome DNA
		2	ATTTAAATACCTGCAGGCATGCAAGCTT	ATTTAAATACTCTAGAGGATCCCCGGGT	pUC-K001
pUC-K003	pUC-ace1	1	CTAGAGTATTTAAATTAACCTGATTTCAAC	TGCAGGTATTTAAATAGAGGTAGTCTAGTC	Genome DNA
		2	ATTTAAATACCTGCAGGCATGCAAGCTT	ATTTAAATACTCTAGAGGATCCCCGGGT	pUC-K001
pUC-K004	pUC-rce1	1	CTAGAGTATTTAAATGGTCAACGGAGGCCAGAAGA	TGCAGGTATTTAAATATTTTGCGCATGACGGGCGA	Genome DNA
		2	ATTTAAATACCTGCAGGCATGCAAGCTT	ATTTAAATACTCTAGAGGATCCCCGGGT	pUC-K001
pUC-K005	pUC-xyr1	1	CATTAATCAGTCACAATGTTGTCCAATCCTCTCCG	TTTCGCCACGGAGCTTTAGAGGGCCAGACCGGTTC	Genome DNA
		2	ATTTAAATACCTGCAGGCATGCAAGCTT	ATTTAAATACTCTAGAGGATCCCCGGGT	pUC-K001
pUC-K006	pUC-crt1	1	CATTAATCAGTCACAATGAAGGAGCCGCCCAAGGC	TTTCGCCACGGAGCTTTAAGCCTTCTCGATATTGA	Genome DNA
		2	ATTTAAATACCTGCAGGCATGCAAGCTT	ATTTAAATACTCTAGAGGATCCCCGGGT	pUC-K001
pUC-K007	pUC-bglr	1	CATTAATCAGTCACAATGACATCGGCCGTCAAGCG	TTTCGCCACGGAGCTCTACCGCAGTGTGTTGAAGT	Genome DNA
		2	ATTTAAATACCTGCAGGCATGCAAGCTT	ATTTAAATACTCTAGAGGATCCCCGGGT	pUC-K001
pUC-K008	pUC-vib1	1	CATTAATCAGTCACAATGACCGACCTCAGAGGAGA	TTTCGCCACGGAGCTTTAATTACCATGCCAGGAGT	Genome DNA
		2	ATTTAAATACCTGCAGGCATGCAAGCTT	ATTTAAATACTCTAGAGGATCCCCGGGT	pUC-K001
pUC-K009	pUC-ace2	1	CATTAATCAGTCACAATGGACCTCCGGCAAGCATG	TTTCGCCACGGAGCTTCACTTCAGCAGTCTGGCAC	Genome DNA
		2	ATTTAAATACCTGCAGGCATGCAAGCTT	ATTTAAATACTCTAGAGGATCCCCGGGT	pUC-K001
pUC-K010	pUC-ace3	1	TAATCAGTCACAATGGCCACAGCGGCCGCGGCAGC	CGCCACGGAGCTTTAGCCAACAACGGTAGTGGACG	Genome DNA
		2	ATTTAAATACCTGCAGGCATGCAAGCTT	ATTTAAATACTCTAGAGGATCCCCGGGT	pUC-K001
pUC-K011	pUC-cbh1	1	CTAGAGTATTTAAATAGCTCCGTGGCGAAAGCCTG	TGCAGGTATTTAAATGGAGCCATACTGGCGGGAAC	Genome DNA
		2	ATTTAAATACCTGCAGGCATGCAAGCTT	ATTTAAATACTCTAGAGGATCCCCGGGT	pUC-K001
pUC-K012	pUC-pyr4	1	CTAGAGTATTTAAATCAAACCAGCCAAGGTAGGTA	TGCAGGTATTTAAATATCACATGTCAATGTCACAA	Genome DNA
		2	ATTTAAATACCTGCAGGCATGCAAGCTT	ATTTAAATACTCTAGAGGATCCCCGGGT	pUC-K001
pUC-K013	pUC-Tr120830	1	CTAGAGTATTTAAATTTTCGCCTTAGCGGTTCCAT	TGCAGGTATTTAAATTTAATACTCGGCAGTCTCGTCAG	Genome DNA
		2	ATTTAAATACCTGCAGGCATGCAAGCTT	ATTTAAATACTCTAGAGGATCCCCGGGT	pUC-K001
pUC-K014	pUC-Tcbh1-Tr120830-3'	1	CTAGAGTATTTAAATAGCTCCGTGGCGAAAGCCTG	AAGCGCGCGGTACTTCTCGGCTACGTTGTCATCGT	pUC-K011
		2	AAGTACCGCGCGCTTGACAA	TGCAGGTATTTAAATCTGAATGCCCGGTGGTAAGC	pUC-K013
pUC-K015	pUC-pyr4-Tr120830-3'	1	CTAGAGTATTTAAATCAAACCAGCCAAGGTAGGTA	CCATCACATGTCAATGTCAC	pUC-K012
		2	ATTGACATGTGATGGAAGTACCGCGCGCTTGACAA	TGCAGGTATTTAAATTTGGTTCTTGGTTTGGAGGG	pUC-K013
pUC-K016	pUC-Tcbh1-Tr120830-3'-pyr4-Tr120830-3' (pyr4*)	1	CTAGAGTATTTAAATAGCTCCGTGGCGAAAGCCTG	CTGAATGCCCGGTGGTAAGC	pUC-K014
		2	CCACCGGGCATTCAGCAAACCAGCCAAGGTAGGTA	TGCAGGTATTTAAATTTGGTTCTTGGTTTGGAGGG	pUC-K015

Table S6 Constructed expression cassettes and primer pairs

Plasmid		Homologus region	Promoter	Gene	Terminator	Maker	Homologus region	Fragment	Forward Primer(5'→3')	Reverse Primer(5'→3')	Templete
pUC-K017	pUC-ΔAce1	*ace1-5'*	-	-	-	*pyr4**	*ace1-3'*	1	CAAACCAAGAACCAAAAAGATTGCGACACATACAA	TTTCGCCACGGAGCTGGCGGCCGAGATCTGTGTTC	pUC-K003
								2	AGCTCCGTGGCGAAAGCCTG	TTGGTTCTTGGTTTGGAGGG	pUC-K016
pUC-K018	pUC-ΔRce1	*rce1-5'*	-	-	-	*pyr4**	*rce1-3'*	1	CAAACCAAGAACCAACATAACTGAGAGAACCAGAA	TTTCGCCACGGAGCTGACTGCCTGCCTCCCAGTTT	pUC-K004
								2	AGCTCCGTGGCGAAAGCCTG	TTGGTTCTTGGTTTGGAGGG	pUC-K016
pUC-K019	pUC-ΔAce1-Pact1-Xyr1	*ace1-5'*	*act1*	*xyr1*	*cbh1*	*pyr4**	*ace1-3'*	1	AGCTCCGTGGCGAAAGCCTG	GGCGGCCGAGATCTGTGTTC	pUC-K017
								2	CAGATCTCGGCCGCCACGGGGTACGCGTGAAATTG	TGCAGGTATTTAAATTGTGACTGATTAATGTATGA	pUC-K002
								3	ATGTTGTCCAATCCTCTCCG	TTTCGCCACGGAGCTTTAGAGGGCCAGACCGGTTC	pUC-K005
pUC-K020	pUC-ΔAce1-Pact1-Xyr1^{V821F}	*ace1-5'*	*act1*	*xyr1* ^{V821F}	*cbh1*	*pyr4**	*ace1-3'*	1	CACGCGTTCTCGGCTGCCGAAGCTATT	AGCCGAGAACGCGTGGCTCGTCGCCGT	pUC-K020
pUC-K021	pUC-ΔAce1-Pact1-Xyr1^{A824V}	*ace1-5'*	*act1*	*xyr1* ^{A824V}	*cbh1*	*pyr4**	*ace1-3'*	1	TCGGCTGTCGAAGCTATTAGCCAGATT	AGCTTCGACAGCCGATACCGCGTGGCT	pUC-K020
pUC-K022	pUC-ΔRce1-Pact1-Crt1	*rce1-5'*	*act1*	*crt1*	*cbh1*	*pyr4**	*rce1-5'*	1	AGCTCCGTGGCGAAAGCCTG	TGTGACTGATTAATGTATGA	pUC-K018
								2	CATTAATCAGTCACAATGAAGGAGCCGCCCAAGGC	TTTCGCCACGGAGCTTTAAGCCTTCTCGATATTGA	pUC-K006
pUC-K023	pUC-ΔRce1-Pact1-BglR	*rce1-5'*	*act1*	*bglr*	*cbh1*	*pyr4**	*rce1-5'*	1	AGCTCCGTGGCGAAAGCCTG	TGTGACTGATTAATGTATGA	pUC-K018
								2	CATTAATCAGTCACAATGACATCGGCCGTCAAGCG	TTTCGCCACGGAGCTCTACCGCAGTGTGTTGAAGT	pUC-K007
pUC-K024	pUC-ΔRce1-Pact1-Vib1	*rce1-5'*	*act1*	*vib1*	*cbh1*	*pyr4**	*rce1-5'*	1	AGCTCCGTGGCGAAAGCCTG	TGTGACTGATTAATGTATGA	pUC-K018
								2	CATTAATCAGTCACAATGACCGACCTCAGAGGAGA	TTTCGCCACGGAGCTTTAATTACCATGCCAGGAGT	pUC-K008
pUC-K025	pUC-ΔRce1-Pact1-Ace2	*rce1-5'*	*act1*	*ace2*	*cbh1*	*pyr4**	*rce1-5'*	1	AGCTCCGTGGCGAAAGCCTG	TGTGACTGATTAATGTATGA	pUC-K018
								2	CATTAATCAGTCACAATGGACCTCCGGCAAGCATG	TTTCGCCACGGAGCTTCACTTCAGCAGTCTGGCAC	pUC-K009
pUC-K026	pUC-ΔRce1-Pact1-PT-Ace3	*rce1-5'*	*act1*	PT-*ace3*	*cbh1*	*pyr4**	*rce1-5'*	1	AGCTCCGTGGCGAAAGCCTG	TGTGACTGATTAATGTATGA	pUC-K018
								2	CATTAATCAGTCACAATGCTGCGCTACTCCCCCGT	TTTCGCCACGGAGCTTTAGCCAACAACGGTAGTGG	pUC-K010
pUC-K027	pUC-ΔRce1-Pact1-FL-Ace3	*rce1-5'*	*act1*	FL-*ace3*	*cbh1*	*pyr4**	*rce1-5'*	1	TAAAGCTCCGTGGCGAAAGCCTG	CATTGTGACTGATTAATGTATGA	pUC-K018
								2	TAATCAGTCACAATGGCCACAGCGGCCGCGGCAGC	CGCCACGGAGCTTTAGCCAACAACGGTAGTGGACG	pUC-K010

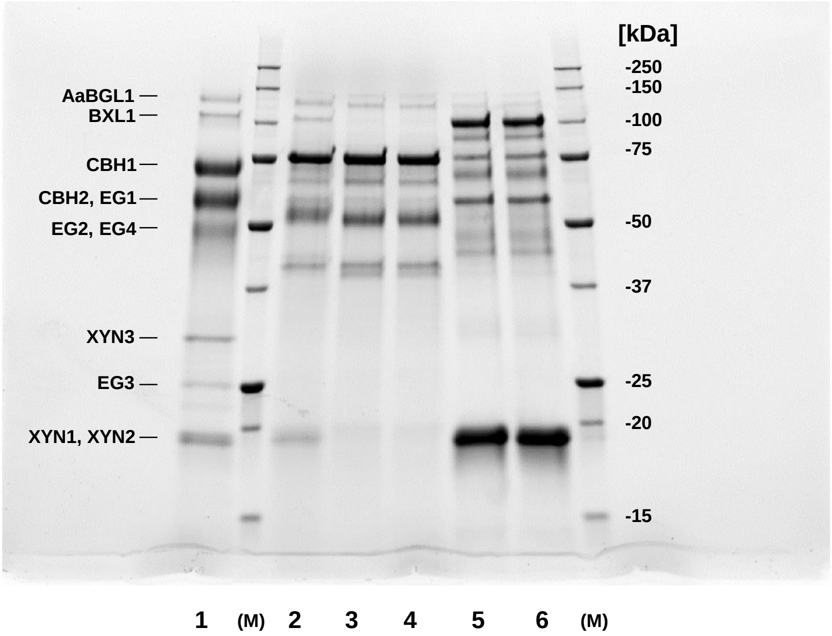

Fig. S4 Original SDS-PAGE gel in Fig. 1b

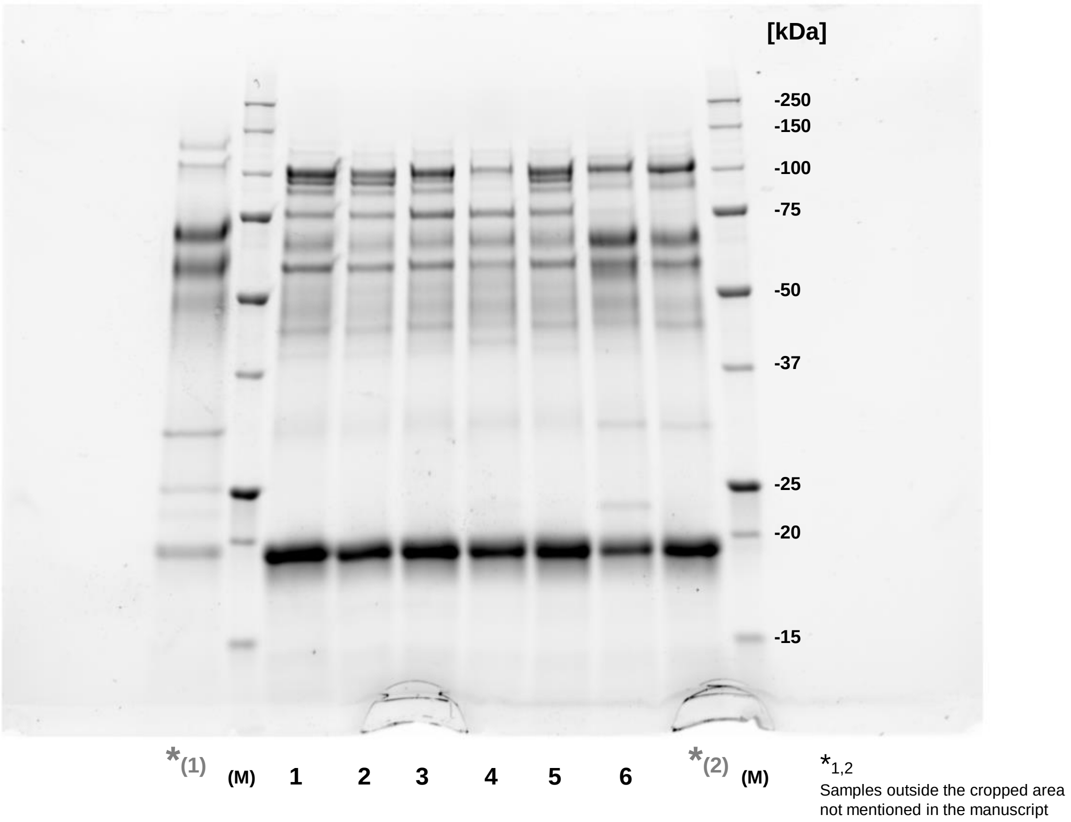

Fig. S5 Original SDS-PAGE gel in Fig. 2b

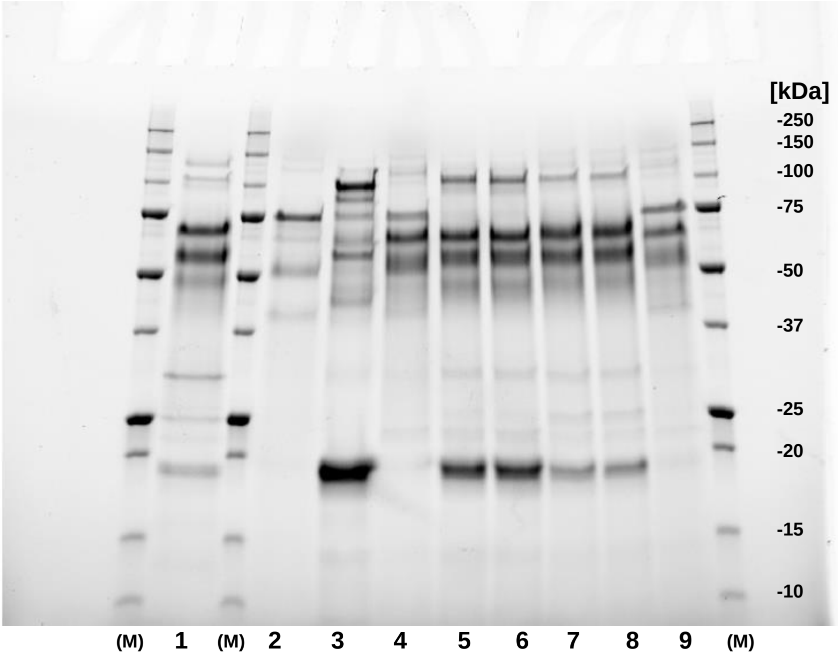

Fig. S6 Original SDS-PAGE gel in Fig. 3b
